# Identifying how classroom teachers develop presence online: breaking the fourth wall in online learning

**DOI:** 10.1007/s10639-023-11714-8

**Published:** 2023-03-22

**Authors:** Sarah Prestridge, Katherine Main, Mirjam Schmid

**Affiliations:** School of Education and Professional Studies, Griffith University, Mt Gravatt Campus 176 Messines Ridge Road Mount Gravatt, Brisbane, QLD 4122 Australia

**Keywords:** Presence, Place presence, Teacher presence, Online teaching, Online design

## Abstract

This paper examines the invisible barrier that can challenge teachers when teaching online, called the fourth wall. Using a presence framework derived from the literature, we explored how experienced teachers manage the absence of visual cues and identify the pedagogical practices they adopted as a response. Data from semi-structured interviews with 22 teachers experienced in online teaching was analysed for individual presence, place presence, and co-presence. Results indicate seven different types of individual presence, four different types of place presence, and three different types of co-presence. Overall, findings show that teachers discussed developing students’ individual connections to the online lesson more often compared with developing co-presence (student-to-student engagement) with place presence being representative of the online learning space. Specific strategies that teachers used to support each presence are presented and implications are provided for how this affects the move to an increased use of blended and online learning in the schooling context.

## Introduction

Shifting to online teaching has been challenging for classroom teachers. Even before the significant and forced shift to online learning due to COVID-19, there was an increasing trend towards online learning in many school settings. Within these settings, teachers faced the challenge of the boundary created by physical distance and the use of communication technologies. Teaching online has created the need for another paradigm shift in how we perceive the teacher-student relationship and understand effective learning in the online space.

Historically, teachers were perceived as the fount of all knowledge and the cliché “sage on the stage” described a teacher-centred, transmissive style where the teacher delivered content to students. The recognition that students had a concomitant role in the teaching and learning process saw the introduction of a constructivist view of learning that has its roots in the work of Dewey (Otto & Dewey, 1931; Bruner, 1961; Vygotsky, 1962; Piaget, 1980). Constructivism is a student-centred approach to teaching and learning that positions the teacher as the “guide on the side” and the student as an active participant in the learning process (Driscoll, 2000). To date, much research has been centred around explaining the characteristics of constructivist learning (Tam, 2000), the goals of constructivist learning (Honebein, 1996), and the benefits of constructivism (Bada, 2015). However, the move towards online learning has introduced another element into the teaching and learning process that has, in many instances, seen teachers revert to a position of “sage on the stage” as they grapple with how to break down the invisible barrier created by the use of technologies (Ellis & Bliuc, 2019; Ewing & Cooper, 2021).

The invisible barrier that is challenging teachers when teaching online can be likened to the *fourth wall*. The fourth wall is a theatrical term coined by Diderot, that refers to the invisible wall that separates the actors on a stage and the audience (Bell, 2008). It can be argued that the technologies used in online learning have become the fourth wall in online teaching. For teachers, a lack of skills in how to engage students has led to the regression in pedagogical practices where, again, they have become the performer (sage on the stage), and the students have taken the role of the audience or spectators. As such, technologies have become a powerful, invisible force that has influenced the behaviour of anyone who enters the “performance” of a lesson. The breaking of the *fourth wall* when teaching online requires teachers and students to “meddle in the middle” and engage constructively together as co-actors.

This study builds on the work of McWilliam’s (2008) call to unlearn the pedagogical habits that are no longer valuable for new communication mediums. By definition, McWilliam’s term “meddler-in-the-middle” represents a pedagogical shift beyond sage-on-the-stage and guide-on-the-side, where mutual involvement in teaching and learning is argued as the dominant requirement for a post-millennial social world. The teacher is referred to as a designer, a collaborative critic and an authentic evaluator. This paper aims to identify the online practices that teachers engineer to enable all actors (teachers and students) to meddle in the middle.

The emergence of this paper and the problem of the fourth wall came from the first round of a thematic analysis of interviews with distance education teachers. Within a study that was investigating the difference between classroom and online (at a distance teaching) practices, a key challenge emerged for teachers when teaching online, that is, not being able to read students’ visual cues. Russo and Benson (2005) have termed this challenge as teaching with “invisible others”. It seemed to the researchers that a solution to the barrier of the *fourth wall* was to examine how experienced teachers manage the absence of visual cues and what pedagogical practices they adopted as a response.

## The challenge of seeing online learning engagement

Online teaching and learning can be challenging for both students and teachers (see Rasheed et al., 2020). Cited challenges include pointing to students’ lack of engagement when online and teachers perceiving that engagement is lost (Carr, 2000). Engagement online can be represented by how the teacher is seeing and/or feeling that the students are present, such as by simply attending the session, putting a camera on, or posting a comment in a chat window; students may work together in a breakout room or on an asynchronous task over a period of time; or the students work independently and complete a quiz/assessment task (Bervell et al., 2020; Prestridge & Cox, 2020). Further examination of these engagements indicates that teacher-to-student and student-to-student-interactions online are vital to build relationships (Alqurashi, 2019); to engage in learning behaviours such as posting, discussing, or chatting (Wu & Hiltz, 2004); and also to support engagement with the material or content (Bervell et al., 2020). The teacher’s role in engineering this engagement is also important as Bervell et al. (2020) found that student-to-teacher-interactions mediate student-to-student-interaction of online content. Researchers have explored the characteristics of the student as influencing online engagement, for example, self-efficacy, motivation, social presence, technology skills, and self-regulation (see, for example, Kim & Glassman, 2013; or Wang et al., 2013). Few authors have looked at the pedagogical strategies that engineer these engagement types.

Some research has examined online teachers’ roles and conceptions of best teaching approaches (see Brinthaupt et al., 2011; Gonzlez, 2009, 2010, 2012). However, these studies reflect adult learning paradigms and provide more generic directives such as to ‘foster student engagement’ by creating a community of learners or using a blog for reflective thinking. These global statements do little for the challenges teachers face with “invisible students”. To add to this predicament, the research done during the COVID-19 remote teaching period pointed to students “pin[ing] to meet online and … always willing to contribute to discussions or show examples of their learning” but could be discouraged by being told to mute by the teacher or being briefly given tasks and then left to complete work independently (Ewing & Cooper, 2021, p. 48). What is needed is a deeper understanding of the development of a sense of presence of both the teacher and the student in an online setting and how the teacher develops this. This paper turns now to understanding online presence.

## Pedagogy of presence

The barrier of the fourth wall in online learning reduces the teacher’s opportunity to read visual cues, which has been evidenced historically through the concept of online presence. In the 2000s, with the emergence of the Community of Inquiry framework, Garrison et al. (2001), purported three types of ‘presences’ for effective online engagement: cognitive presence, teaching presence, and social presence. These three presences were grounded in a student-centred model (Kozan & Richardson, 2014), emphasising critical thinking and collaboration. Notwithstanding the fact that the three presences are co-dependent, there seems to be a rationalisation for the development of social presence, that is the development of a community of learners before the enactment of teaching presence and cognitive presence. Cheney and Bronack (2011) suggested a focus on a ‘presence pedagogy’ when online, as this approach foregrounds social engagements of users but also facilitates the meaningful engineering of student-to-student-interactions while maximising collaboration. In other words, these researchers and others are purporting that social cohesion needs to exist before critical-cognitive engagement can occur (Alqurashi, 2019).

There is evidence that social presence matters. Borup et al. (2012) examined the use of videos accessible asynchronously and found that social presence led to improved student participation with this content. Russo and Benson (2005) suggested that students’ perceptions of the presence of other students and the instructor in an online class are significantly related to students’ positive attitudes and their level of satisfaction with their own process of learning. Further, Picciano (2002) observed that online students’ sense of being related to the presence of others, that is, co-presence, with peers or their instructor/s and affected their perception of their performance and growth. It seems that a student’s own presence and the presence of others within a communal space, that is a place presence, are contributing elements of social presence. Bulu (2012) has explored these three presences: social presence, co-presence, and place presence, finding that social presence seemed to affect satisfaction the most, while students’ perception of place presence and their co-presence all strengthen social presence.

An examination of these presences is needed as much of this work draws from a higher education or adult learning context. Within the context of schooling, however, these presences need closer examination as the teachers have a heightened responsibility to support the creation of these presences. Building on the work of these researchers, situating the research in the schooling context, and also adopting the position of the teacher being responsible for developing presence, this study frames an analysis of online pedagogy using three presences: individual, place, and co-presence.

## Theoretical framework of individual, place and co-presence

Social presence has been originally defined as “the ability of participants to project themselves socially and emotionally, as ‘real’ people (i.e., their full personality), through the medium of communication being used” (Garrison et al., 2001, p. 94). This is evidenced online through a person’s expression of emotion as well as reciprocal and respectful communication exchanges (Garrison et al., 2001). From this perspective, social presence is an individual construct shaped by their connection to the online group, class, or community. In this sense, social presence is represented by how the individual feels connected to the group through their self-expressions. For this study, it will therefore be phrased as ‘individual presence’.

Place presence refers more to the influence of the actual space in supporting a person’s sense of presence (Gunawardena, 1995; Kamada et al., 2005). Parker et al. (1978) proposed that a communication medium could influence the intimacy and immediacy, and thereby, the way people interacted within it either more or less personally. Social interactions also influence place presence by enabling greater quality and capability of the communication medium, such as from written, text-based media to face-to-face media. The concept of being in an online place, “somewhere”, such as on a Zoom meeting or on the class OneNote page, represents place presence.

Co-presence is more relative to others. Goffman (1956) defined this sense of presence as a sense of being together in a virtual environment where individuals become “accessible, available, and subject to one another” (p. 22). Co-presence renders the teacher/learner uniquely accessible, available, and subject to one another. Schroeder (2007) further helped to separate the meaning of individual and co-presence, where the former is the subjective experience (the user’s self-report), and the latter is the objective measures (task performance, action in the task/activity). In their study of presence, Parrish et al. (2021) found that online students perceived presence as “teamwork” which aligns with Schroeder’s task-oriented construct.

Individual, place, and co-presence represent a learner’s perception of presence online. To examine the development of presence by the teacher, however, it is necessary to draw on further frameworks and theories within online and distance education. The types of interactions espoused by Moore (1989) and Hillman et al. (1994) provide some understanding of what presence may look like online pedagogically.

Moore’s (1989) classification of interactions aligns with individual presence and co-presence. ‘Learner-to-content’ interaction is a self-directed, individual form of learning where the learner interacts with the content provided by the teacher and through this engagement develops a sense of individual connection to the group/online class. Moore’s other two forms of interaction, ‘learner-to-instructor’ and ‘learner-to-learner’ interaction, are instructional and dialogue based as tasks or activities that are part of the online class. Hillman et al. (1994) introduced ‘learner-to-interface’ interaction that focuses on the technology as an intermediary between the student and the content and the other learners. Drawing on these representations of presence in the literature, alignments can be made between types of online interactions and presences (see Table 1). This presence framework is provided to guide this study.

**Table 1:** Presence Framework

Type of interaction	Presence
Learner-content: interaction occurs when the student intellectually engages with content and expressing it in some way resulting in a feeling of connectedness to the group/class	Individual presence
Learner-learner-instructor: interaction occurs between the students, alone or in a group and or with the teacher	Co-presence
Learner-interface: interaction occurs with a focus on the technology as an intermediary between the students and the content	Place presence

**Fig. 1:**
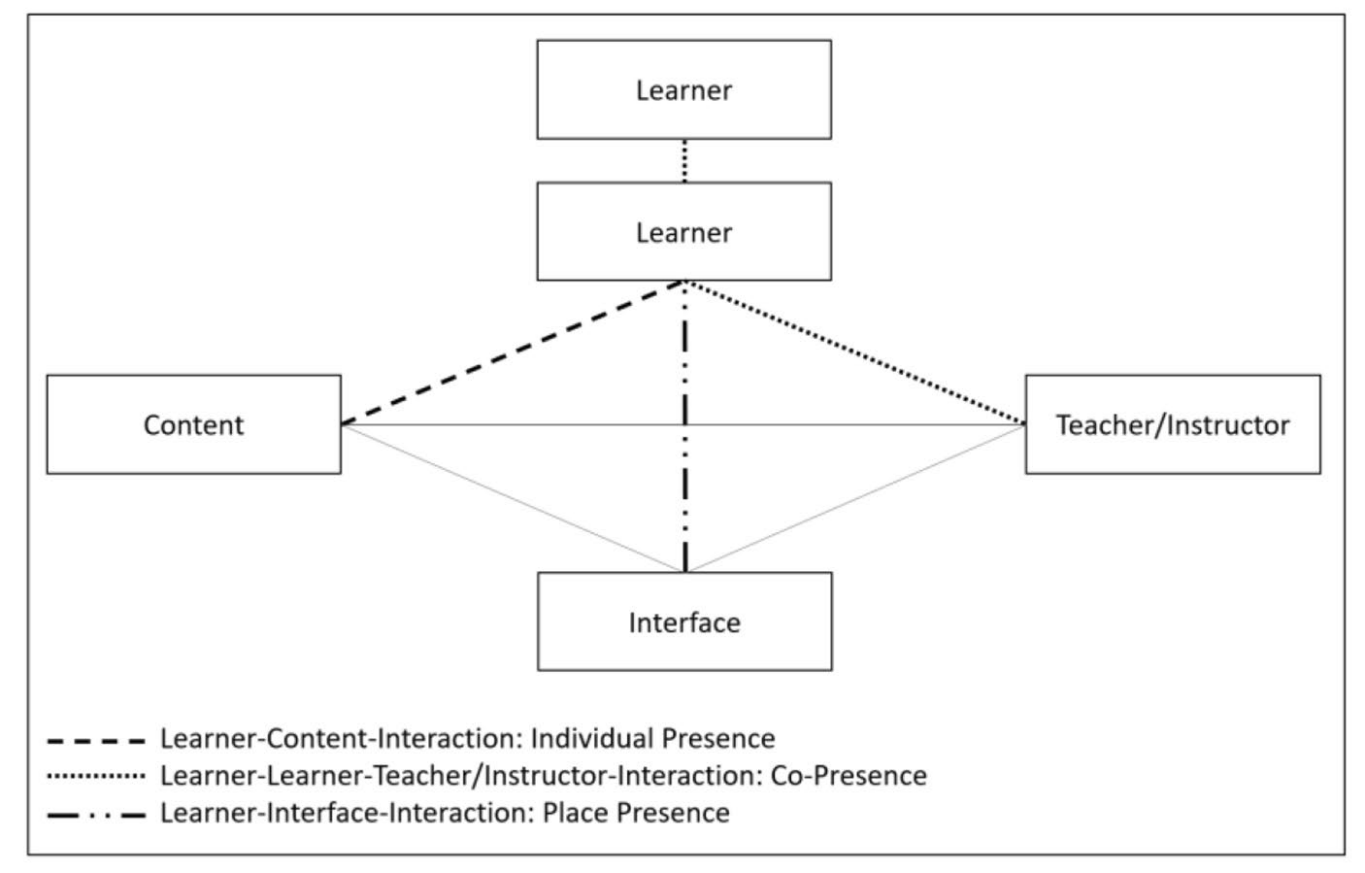
Different interactions and Presences

As described by different authors, there are interactions between the different “elements” (i.e., learner, content, teacher/instructor and interface). In our study, as described above, we are interested in three presences that result from the interactions (connected with different dotted lines; see Fig. 1) and specifically how the teacher engineers these types of interactions to develop the presences.

While presence does matter, there is still a lack of understanding of how to effectively establish it online, especially in the newer context of K-12 schooling (Borup et al., 2012). To date, research based on student perceptions has dominated with little to no research informed by classroom teachers’ practices online at a distance. This study approaches the problem of engineering presence in a more grounded and exploratory manner through the simple but complex pedagogical examination of *How are school teachers creating presence online*, which we believe will break the fourth wall.

## Method

### Sample and data collection

To explore the different presences in online teaching, twenty-two (*N* = 22; 17 females, 5 males) experienced online teachers participated in semi-structured interviews (see Table 2). On average, teachers had nearly five years of experience in online teaching (*M* = 4.8, *SD* = 3.06, span 1 to 12 years). This study involved a targeted selection of five distance education schools in Australia. These five schools offer fully online classes for students in rural and remote areas, as well as for students who cannot access a regular, face-to-face school setting for health or other reasons. Volunteer teachers received the questions in advance to prepare for the interview.

Each interview included questions about the teacher’s teaching background; beliefs about teaching fully online; how to teach online, specifically what a lesson looks like; their confidence and strategies when teaching online, as well as the difference they see between the classroom and online teaching. The objective for the interviews was not to compare responses teachers gave but, rather, to elicit deeper and more authentic understandings of the emotionally loaded and complex task of teaching online (see also Oppenheim, 1992).

**Table 2:** Participants

Teacher	Subject	Teacher	Subject
Andrew	Junior secondary maths/science	Kimberly	Biology and science
Beryl	Teacher librarian/technology	Lucy	Deputy principal primary
Cheryl	Lead teacher-ICT specialist	Maddy	Numeracy and literacy
George	Mathematics and technology	Maria	Primary teacher
Heather	Primary STEM program	Matthew	Physical education, science and social studies
James	Primary teacher	Natalie	Primary teacher
Jenny	English and history	Patricia	Primary lead teacher
Jessica	History and geography	Rita	Mathematics and coding
Kale	Critical & philosophical thinking, debating	Rosey	Primary teacher
Kathleen	Pedagogical coach/art	Sharon	Primary teacher
Kelly	Music and legal studies	Susie	Japanese teacher

### Development of the presence framework

Interview data were analysed through a thematic coding approach (DeCuir-Gunby et al., 2011) using both, a deductive and an inductive categorising methodology to create a codebook. The presence framework and the three types of presences (i.e., individual, place, and co-presence) were defined based on theory and designated as the three main categories (see Table 3) of the codebook. The following description documents the analysis process to establish the subcategories within each presence in the codebook. These subcategories were considered the strategies teachers used to establish the type of presence.

**Table 3:** Presence framework definitions

Presence type	Description
Individual presence	Teacher supports the student to develop a sense of belonging to the online class/course
Place presence	Teacher supports the student to connect to the online space, digital tool, online environment
Co-presence	Teacher supports the student to work together

In the first round of analysis five random interviews of the 22 interview transcripts were used by two researchers to gain an overview of the data and to create subcategories. This analytical process can be described as an inductive content analysis process in concert with the deductive process (see also Mayring, 2015). Specifically, the two researchers jointly analysed the first five interviews to create the codebook. In a process of negotiation about each category, they formulated subcategories and their descriptions and looked for suitable anchor examples. It was decided that each interview excerpt was allocated to only one subcategory.

The two researchers separately coded a random interview (Susie). They then discussed this transcript and coded in detail with each other and adapted some of the subcategories. Next, the same researchers coded two more interviews (Andrew and Lucy) separately, applying the new codes and adding further categories where necessary. Subsequently, the researchers met again and discussed the coding with each other. In doing so, they further specified the categories and their definitions and also identified other anchor examples. Following this, the researchers used the adapted codebook to code a fourth interview (George) separately and then again discussed these categories with each other. At this point, a third researcher independently analysed an interview (Sharon) and discussed the established categories with one of the researchers. As the codebook was found to be understandable and applicable, it was now considered established. The remaining interviews were analysed by two researchers separately. Any unclear passages and codes were discussed together.

## Findings

The need for an examination of presence is highlighted by a comment by one participant, James. In his comment, James explains the problem teachers face when teaching online:

James: It’s just little, I mean at the end of the day you’re trying to, there’s sort of a wall to some extent, between the student and the teacher. So, we really try hard to put that effort in, I guess, to break down those barriers. The whole effort conquers distance kind of slogan, comes into play there. Yeah.

The results of our exploration of *How are school teachers creating presence online*, which we believe will break this *fourth wall* will be presented firstly with the codebook (Table 4), then with a more specific analysis of each presence: individual, place, and co-presence.

**Table 4:** Presence Codebook

	Subcategory	Description	Anchor example
Individual Presence	Connect student to class and content	At beginning of lesson doing something that is fun, attention grabbing to connect the individual to content & others	ensuring that those kids are engaged and ready for learning and are the cued in. Making sure, and I think that’s really important at the beginning of it, that first five, or how we are getting them to engage with us. Kids come in with a lot of baggage, teachers come in with a lot of baggage, and how do I get them ready to engage with what I want them to do, and how am I getting them caught in, how am I getting them to buy in? How do I get them to buy into what I want them to do
	Differentiation/ personalisation	Supporting individual needs	I know the students who are likely to be pretty confident, so I might send them off to do some stuff by themselves, and then the students who aren’t so confident, I might get them to stay in the main room with me, and we might do some more, maybe together
	Task-Authenticity/ related to self	Making a link to the student’s context (real life)	looking particularly at how it relates to me, like self, my community, globally, does this actually affect what’s going on in the rest of the universe
	Emotional response	See/feel/Jokes/Emoji	whether they’re having trouble with something or need a bit of help, so you really rely on them to communicate that to you in some other way, maybe using an emoticon, or putting something in chat, or asking for help, and some of our students don’t do that, so that’s very difficult.
	Teacher presence	Being real, radio voice (teacher performance)	That trick is, I think you need to have the radio voice, and definitely a presence, and they know that you are there, and that you actually take an interest in what they do. It’s really hard if you think you can just turn up and do it and leave, I think they feel that, they know that.
	Relationship building	Getting to know the students personally, building a relationship, - (re-use info)	Whether it’s becoming a personal thing, I’ve made it personal for them and they go, “Oh, I like it, that’s about me,“ and they engage, or maybe it’s more about being really interesting, “Oh, I love birds,“ and I know that about them, and that’s that relationship that I’ve built with them.
	Active involvement	Involve students with some form of active task to do	I want to see what they’re doing, because it’s often the only chance you get to see what they’re doing, other than the assessment task, so I want them to be showing me something on the board. So I think practice in online lessons is important, but it needs to be practice that teachers can see
Place Presence	Online places	Teacher describes some tools as an online place to go somewhere, to put students, as a safe place, e.g., a breakout room, a chat	at least they understand what’s in my universe, it’s in my safe place. Regardless of whether that’s online or in a class, I think in line it’s really important, because we don’t understand that they’ve fought with two people on the way in, so how do I engage them and catch them, and they’re ready to engage?
	Turn-on-space	Once lesson starts student is ‘turned on"… going live	Yeah, it’s also a ritual, because I sit down, I then do my little ritual thing and I put my headsets on… I’m now listening online to the woman say, “If your headset is ready,“ you know doing your headset setup and I’m listening online now, I’m not actually … It is, it’s like a set of blinkers, because then it’s here.
	Design of place	Design of the actual online place/instruments (Description: all written down: 1,., 2.,, here is the link, instructions)	it’s all written down, “Step one, step two, step three, I want you to do this, then this, and then that then we gradually take it off to a visual clue, Here’s the link, that’s the site, this is it.“ That really helps those kids who struggle going, “Oh, what was I supposed to be doing?“ It’s right here, I know where your eyes are, here’s the instruction.
	Personal place	Giving students a place for their personal representations (photos)	put up the photos about them and their dog and their cat and their kitty, and we all comment about it, and they get that nice little personal buzz hit when they get some information from us back, “Really liked your picture, very impressed to see your puppy there.“
Co-Presence	Co-engagement (social)	Students being social with each other (not content-related). Student-student	somebody who’s going to do a private chat to his mate. We tell them too, I’m in God mode, I can see everything, and they all go, “Oh, can you?“, and they’re writing down, “Where are you going at lunch time.“ You go, “Obviously finished,“ you know like, “How about you do this?“, and they’re, “How did she know that?“ They all think that nobody else can read it or see it.
	Co-engagement (academic)	Teacher organising student engagement with each other and the content	the students can then teach to somebody else, for example and part of that some of the students that are quite high, even in the practicing stage even when I’ve modelled and they’re sort of doing it together, some of them actually ask if they can do that one and model it to the class, and yeah, give them that opportunity to do that as well.
	Parents/tutors as co-workers	Parent in various roles: Admin, management, organisation, education role	you really need to keep in contact with the parents and the students more so than you would at mainstream so whenever I email out information to the students, say nights whatever or anything having to do with science, like you need to have this resource for your next lesson. We always put the parent in as well so they both get the message

### Codebook with categories, definitions and anchor examples

The findings indicate that teachers use specific strategies to develop the three types of presence. Individual presence is developed using seven different strategies. These are (a) connecting student to the class and content; (b) differentiation/personalisation; (c) task authenticity/related to self; (d) emotional response like using ‘see-feel-jokes-emojis’; (e) teacher presence; (f) relationship building; and (g) active involvement. This type of presence, that is, developing an individual’s connection to the online lesson, had the most types of strategies. Teachers, therefore, indicate that it is important to create a direct connection to each student when teaching online.

Place presence was based on Hillman et al.’s (1994) ‘learner-to-interface’ interaction. In this study, teachers were creating an online place or space for learning, much like going to a classroom to learn. Within the category of place presence, four different strategies emerged. These were categorised as (a) online places; (b) turn-on-space; (c) design of place; and (d) personal place. In the first subcategory of ‘online places’, the teacher discussed the digital tool as an online place, such as ‘go to the OneNote’ or ‘respond in the chat’. The second subcategory of place presence strategy the teachers identified was the processes involved in helping to switch learning on for the student, such as turning on the microphone or putting on the headset, or logging on at any time. This subcategory was called ‘turn-on-space’. Design of the place was the third subcategory. Teachers in this subcategory discussed the features of the lesson such as *chunking information*, *modelling and practicing*, or a *sequence of visual materials*. The final subcategory for place presence is enabling personal representations such as a photo wall or a student’s own page in the class portal.

Developing co-presence, the third type of presence, focused on students’ engagement with each other and the teacher/s. This category was based on Moore’s (1989) learner-to-instructor and learner-to-learner-interaction. There were three ways this emerged in the data. The first subcategory describes the strategy for students to engage with each other socially (co-engagement social). Some of the strategies described include: chatting before the lesson starts or talking about other things in breakout rooms. The second subcategory was talked about the most by teachers (see Table 5 and further explanations below). In this subcategory, the teacher focused on connecting students to each other through the content (academic co-engagement). The strategies described included: discussion of scenarios, writing comments on what others have written, and sharing with others in the class. The third co-presence focused on connecting to the home, in this case, the parent or tutor as part of the learning network (called Parent/tutor as co-worker). The strategies described included: an after-lesson email or a lot of communication with parents.

**Table 5:**
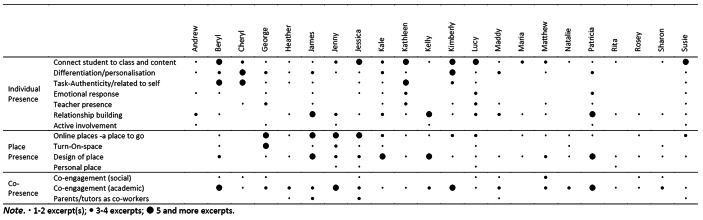
Preference for a type of presence

Overall, findings indicate that teachers discussed developing students’ individual connections to the online lesson more compared with developing co-presence (student-to-student engagement) with place presence being representative of the online learning space. These findings will now be explored in more depth using teachers’ conceptualisations of presence.

### Teachers’ conceptualisations of presence

When coding the interviews, it became clear that teachers were discussing a specific type of presence more than the others. For example, the first analysed interview (Susie) focused on individual presence by *connecting students to class and content*. In this way, Susie kept coming back to talk about this subcategory by describing many different strategies for connecting each child to the content and class throughout the interview. We categorised six interview excerpts within this subcategory for Susie. Interestingly, Jessica did the same thing, but more often than Susie. Jessica referred to this subcategory *connecting students to class and content* eleven times in her interview. It seemed to dominate her thoughts. Another example was George. George talked a lot about place presence, specifically, the subcategories of an ‘online places’ eight times and ‘turn-on-space’ five times. Kale talked about the place presence subcategory of ‘design of place’ twelve times. The data highlighted that teachers had a preference for a type of presence. The following Table 5 presents the number of excerpts a teacher talked about each subcategory. A scale was used: no circle indicates 0 excerpts; a small circle indicates 1–2 excerpt(s); a medium circle indicates 3–4 excerpts, and a large circle indicates 5 or more excerpts.

Teachers’ preference for breaking the fourth wall with a type of presence using specific strategies (subcategories) is evident. However, some teachers hardly or only very briefly addressed how they establish any type of presence (for example Sharon, Heather, Maria, Rita, or Rosey) while all teachers made statements about every type of presences with the exception of Andrew, who only discussed individual presence. Almost all teachers mentioned the following subcategories: ‘connecting students to class and content’ (with one exception: Rosey), ‘relationship building’ (exceptions: Cheryl, George, Natalie), ‘design of place’ (exceptions: Andrew, Cheryl), and ‘co-engagement academic’ (exceptions: Andrew, Maria).

### Unpacking the main types of presences

In this section, we explore in depth the main strategy in each of the presences. These are connect student to class and content for individual presence; design of place for place presence, and co-engagement (academic) for co-presence.

### Individual presence: connect student to content and class

As mentioned above, every teacher except one discussed trying to connect the student to the class and content. In total, there were seventy-two excerpts coded (the largest category in the data) with six teachers discussing this strategy five or more times even though the interview covered a number of elements. For one teacher (Jessica), it dominated her discussion. Connecting the student to the class and content was conceptualised at an individual level.

It was reported that it is important to focus on the individual connection. Many of the teachers suggested that it had to be at the start of a lesson or the start of a new topic when there is a heightened importance to connect the individual to both the (new) content and to the class. For instance, Jessica explains: “*like brand new history or something like that. We do meme collages, so the kids would jump on Google Images and I’ve given them the terms that they need to Google. I say ‘Find a meme about this. Find a meme about that.’ So, you give them a fun little thing to do where they’re just playing, really, but getting something out of it at the same time, a learning experience.*”

The type of activity needs to be generational. As Jessica explained, *“ kids love making memes”.* She related it to the Y-generation within popular culture: “*I do try and make it as pop-y as possible*”. If it is not specifically relevant to social media used by the age grouping, then she believes students disengage quickly. Kathleen, an online Art teacher, also uses this approach to connect students to the class and content: “*The kids love talking about selfies, and this is an installation piece that was ruined by a girl who was having a selfie taken….Something controversial? Someone’s forged something, or something’s been stole”.*

The curriculum area influences what the teachers choose to use to support individual presence. For Jessica, who teaches History, the meme activity can be easily applied to ancient Rome. Similarly, Kathleen the Art teacher, uses a media photo of the girl taking a selfie which ruined an installation piece. She specifically picks controversial images to discuss what interests both boys and girls, as she believes they may not like doing Art. Jessica also teaches Geography. However, social media does not apply as easily, so she uses technologies instead: “*in geography when we first start, so I’m always trying to bring something new in to try and make it a bit more interesting, to try and look at Minecraft, and look at Google Earth”.* Regardless of the subject, Lucy explains that it must be ‘dippy’ which she defines as “*that frontal, you know the dangerous, important, interesting type of activities”*. This suggests that the curriculum area and the age of the student influence the type of activities used to connect students to the content and class, more likely at the beginning of the lesson.

The first thing that is done online seems to be when strategies are needed to connect students individually to the content and class. Both Jessica and Lucy state that it is needed in the first five minutes. Jessica believes this is important because: “*If we don’t capture that right at the start we’ve lost our kids for the next hour, we’ve got such limited time with our kids, we have got to be … get them on board and get them with you”.* Lucy follows this with the idea that: “*Kids come in with a lot of baggage, teachers come in with a lot of baggage, and how do I get them ready to engage with what I want them to do, and how am I getting them caught in, how am I getting them to buy in?”.* Susie also suggests that it is important to create that routine with the warm-up task and that it has to be active in some way: “*Because we know that if we’re not getting them doing when they’re on air, they’re zoning out”.*

There were other approaches described by the teachers, but again these occurred at the beginning of the lesson. These approaches were not ‘activities’ as such but could be considered as chat sessions. Susie described students sharing things at the beginning of the class in a ‘chill and chat session’ such as drawing on the screen or showing a photo of newly hatched chickens. Beryl, on the other hand, asked questions and commented that *getting feedback from the kids about am I on the right track in thinking that this is how you view this*. Her reason for doing this was to ensure that what she was teaching was *relevant to the kids’ needs.*

When connecting the individual student to the class and content, Kimberley explains that when you are online, it is more important to help students *understand why am I doing this at this point in time* and Beryl confirms this but using a multiple sensory approach, in that she says it, writes it and has an icon to prompt as a reminder on her lesson PowerPoint. She states: “*If I wanted to make connections with themselves or with the content or whatever it is, I’ll have it as a jogger in the corner, the little icon. I’m making it really explicit”.*

In summary, one of the ways to create individual presence online was for teachers to use strategies to create a connection between each student and the content and class. These strategies were age-related and usually done at the beginning of the lesson to ensure interest and to motivate engagement. The curriculum area defined the content and as such, influenced either the resource or digital tool used. Alternatively, teachers sought feedback or used more social activities to connect and motivate. Teachers also indicated that they alerted students to the process of making a connection and motivating so that they knew *why* they were doing something and not just *what* they were doing.

### Place presence: design of place

All teachers except two mentioned aspects of the design of the place for online teaching. Overall, there were sixty-five excerpts coded in this subcategory with one teacher, Kale, discussing the design of place twelve times (the most excerpts by a teacher). The design of place refers to how the digital tools and environment are used and structured in a lesson.

Overall, the teachers talked about limited time and therefore, everything needed to be carefully selected and structured. James called it “*precise in what you are teaching for that one hour”.* There are considerations for the digital resources used in the lesson, such as videos, photos, a slide, OneNote page focusing on visual materials plus digital tools to engage students so they were doing things. Kale explains that online: “*You’re essentially putting things in front of them and then changing them with other things. So it’s always going to be a sequence of visual material for the students to work through, which includes multimedia, of course”* and that “*you need some ammunition. You need some tools to keep them engaged”*. Essentially, it is the design of content with the activity.With a lack of visual clues, online teachers talked about chucking information to keep the momentum to the lesson and to keep students active. Kale explains: “*So they’re at a great need then to present kids with a chunk of information and then have activities that are quite … that are sharp activities to get them to work … working”.*

Building on from this and drilling down to the slide design, for example, teachers are designing the slide for flow of the information. Lucy explains: “*There’s tricks with slides, like making sure that you know where their eyes are supposed to be … and you put a slide up or a page up, and it should be really clear where their eyes start and finish, it’s like walking through a painting”*. She warns that you cannot overpopulate a slide, that the design of it is really important to engagement. It is important to know where their eyes are because, as Lucy says, “*It’s like in the classroom, if they’re all looking out the window, they’re not looking at you, they’re not paying any attention”.*

Design of the place also includes asynchronous activities that students can do after the lesson. Kale called this ‘space learning’. He explained that this was where students could finish tasks, but as a teacher, he did not feel like he had any control over students actually doing the activities, but he could influence their engagement by linking it to the next lesson. There was also the consideration that asynchronous activities are only taken into account by the students as homework. Kelly explains that this can impede engagement. She designed a discussion board activity to occur asynchronously as she thought anxious children might participate more. Kelly explains that “*As it turns out, I think the problem that I’m having is that it feels like homework. And so they’re loathe to do it”*. She has found that there is a reluctance to ‘*volunteer thing’* unless you make it part of an assessment or a requirement. She wanted to get students going through content asynchronously and using synchronous time for discussion. She created a lesson package: “*We call it a lesson package where it’s basically the content delivery. And they’re supposed to watch it before they come to class. There’s little quiz questions and all of that sort of thing that they work through”.* She explains that she has ended up re-teaching it in class time and that making it an assessment is the only thing that will get students to do it.

In summary, to create place presence online, teachers designed highly curated lessons that included multimedia materials with activities in a sequence that kept students engaged and doing things. The design of the slide presentation is also important to help the student follow through the visual and written materials, likened to walking through a painting. Teachers also think about the relationship between the synchronous lesson and asynchronous activities. They have designed these to be relational, but students currently perceive asynchronous tasks as homework which means that it is not usually completed. Making it a requirement and linking it more to the synchronous lesson is a proposed solution at this point.

### Co-presence: co-engagement (academic)

Co-engagement (academic) was the largest subcategory in co-presence. This subcategory refers to the teacher engineering students’ engagement with each other within an academic task. Compared to the other subcategories above, *connect student to content and class* which had 72 excerpts and *design of place* which had 65 excerpts; *co-engagement (academic)* was not discussed as frequently with 60 excerpts. Four teachers mentioned aspects of co-engagement (academic) more than five times.

Beryl raises an important point when talking about students engaging together online. She stated that “*there’s an assumption made that because these kids all learn online, they’re aware with how to behave in online learning communities and how to participate in social learning communities as well, and they actually don’t really”.* This was mentioned as the reason for a more structured design for the lesson (in the previous section) as students need scaffolding on what to do and how to do it. This scaffolding was described by Kimberley: “*But generally then an activity where they can share ideas and help each other through progression of questions”*. She believes that it requires high scaffolding but getting students to work together means that they are exposed to different perspectives and backgrounds and it generates more ideas.

Generally, teachers used breakout rooms for students to collaborate on group tasks in synchronous lesson time. Whiteboards or, as George calls them, *think boards*, are used with the whole class for brainstorming activities. Chat is also available for students to ask questions. However, depending on how the teacher uses it, it can be more directed at the teacher than other students. George, for instance, described a strategy to build students’ skills to give constructive feedback, such as, “*We always talk about either constructive or positive. I like the way you, or have you considered, that sort of thing. The kids love that”*. To set this up, George asks students to look at others’ work, then reflect on their own effort and then improve on what they have done. Through this process, students have “*transformed in a little way during that lesson just because they’ve had the opportunity to have a look at someone else’s thinking”.* Interestingly, George believes this process is quicker and easier online as there is no noise disruption from moving around like in a classroom.

There was evidence of the use of asynchronous engagement. Beryl describes an activity where she engineered students sharing and discussing a passion project that inspired them using a wiki space. She believes these kinds of activities are a necessary part of *building community* and they need to happen anytime asynchronously because when they find the inspiring project, they need to share it right then: “*If they didn’t have that setup in that space for them to access whenever they wanted to access, it’s not like they’d go ring up their friend and go, Oh, guess what? I found this person who did this inspiring thing, and I think you should really look at it”.* James also used a wiki to get students to comment on chapters when writing narratives, “*Their view, or the questions, and the other students could see what was posted. I really liked that”.*

In summary, to create co-presence academically online, that is, engineering student-to-student engagement with and around the content, teachers used online group work that was highly scaffolded. They identified that students do not know how to work in a group online and or learn within a community and that this is not part of their generational knowledge. Teachers used tools during synchronous lessons, such as shared whiteboards and breakout rooms but also set up spaces asynchronously so that students’ contributions and discussions/comments could occur at flexible times.

## Discussion and conclusions

Since the 2000s, there has been an agreement of the importance of presence with the conception of the Community of Inquiry framework (Garrison et al., 2001). This study goes beyond that acknowledgement that presence matters when teaching online and provides a more representational view of both the types of presence that teachers are developing as well as their preference for those types of presence and associated strategies. In answering the research questions: *How are school teachers creating presence online*, which we believe will break the fourth wall, the examination finds that teachers put greater emphasis on building individual presence, mainly through strategies which connect each student to the class and content. There is an emergence of place presence, such that the online tool or environment becomes a place for learning much like a classroom and the design of a lesson takes into account content curation and representation, structure and delivery. Engineering students’ engagement with each other around the content is important but not a priority compared with individual engagement.

There were specific strategies that emerged within each presence. For the development of individual presence, teachers used motivating tasks to connect each student to the content and the class, usually at the beginning of the lesson. For place presence, teachers designed highly curated and structured lesson materials and sequences of content with activities aligned to the level of information flow on slide presentations. For co-presence, teachers used group work online in breakout rooms and also asynchronous tools for anytime engagement. However, they indicated that learning with others online needed a lot of scaffolding.

There are two major contributions that can be drawn from this study of presence. First, individual presence may be needed before co-engagement can be engineered. Teachers in this study identified the importance of building each student’s connection to the online course/community as foundational to learning online. Moore’s (1989) classification of interaction identified student-to-content, student-to-student, and student-to-teacher as part of the discourse dynamic, which Bernard et al. (2009) purport to improve student achievement outcomes. However, as found by Ewing and Cooper (2021), teachers are more able to facilitate students’ engagement to content compared to students’ social and cognitive engagement. This finding may support the claim for a developmental view of presence from individual to co-engagement, with further examination of the interrelationships of place presence. In other words, creating a place and connecting the individual to that place (content and class) foregrounds students’ readiness for co-engagement.

Secondly, across all three presences, there was the emergence of the need to build student competencies *for* how to learn online. This is considered to be the thread that weaves through the presences. How to learn was described by the teachers through the presences as *why, what, and how* to do a task or engage with content/others. To support individual presence, teachers reasoned why the students had to do an activity, why it was relevant and why the task was required, place presence required highly curated designs of sites and presentation slides, and co-presence was highly scaffolded group activities. These strategies were building students’ independent learning capabilities often referred to as self-regulation skills which have been evidenced as critical to online learning, namely, effort regulation, time management, metacognition, critical thinking (Broadbent, 2017), and peer learning (Lim et al., 2020). Clearly, a key component of online teaching is teaching students the self-regulation skills necessary to learn online.

## Limitations and future research directions

The findings in this study are contextualised to the wholly online setting. Further studies will be needed to explore whether these findings of presence and associated strategies can be applied to new models emerging in schools post-COVID-19. For example, blended modes of learning that use both face-to-face classes and online learning spaces or hyflex teaching (highly flexible teaching), where the teacher is teaching students in a classroom and online at the same time. Key to understanding whether these findings can be generalised across all online and hybrid settings is dependent also upon how the teacher builds the relationship between online and classroom teaching spaces.

In this study, it was evident that synchronous activities were like classroom teaching (in real-time) and dominated the learning paradigm with asynchronous activities of lesser value and equivalent to homework. However, for online learning to be a valuable element of the learning paradigm, this relationship needs to shift. It is suggested here that for online learning to be valued within a classroom context (as in blended), the relationship needs to shift from the classroom being the dominant learning space with increasing use of learning activities (not revision activities) occurring asynchronously. This shift would then optimise the capabilities that online learning provides, which is a networked system of user engagement (Prestridge et al., 2021). This online networked learning system orientates to users, that is students, engaging in many-to-many communication practices where the teacher is one of the co-participants.

Teachers need to combine different areas of knowledge to teach effectively in the 21st century (see e.g., Technological Pedagogical Content Knowledge-framework by Mishra & Koehler, 2006). Teaching online is one of these areas. Currently, in an online classroom and in models of hyflex and blended, teachers need strategies to break the fourth wall by establishing presence as a place for learning online where each individual is connected so that they are able to connect to others effectively. Therefore, these findings give us a pathway forward and could also be used in the education and training of teachers.

## Data Availability

The datasets generated during and/or analysed during the current study are not available due to the nature of the data and the ethical consideration of participant anonymity. There is no supporting data files/access.
